# Phase Transition Behavior and Threshold Characteristics of GeTe Thin Films Under Single-Pulse Nanosecond Laser Irradiation

**DOI:** 10.3390/ma18235466

**Published:** 2025-12-04

**Authors:** Yajing Li, Xinyu Ma, Qiang Chen, Sixian Qian, Yixuan Jiang, Yuejun Zheng, Yunqi Fu

**Affiliations:** College of Electronic Science and Technology, National University of Defense Technology, Changsha 410073, China; liyajing23@nudt.edu.cn (Y.L.); qsxnudt@nudt.edu.cn (S.Q.);

**Keywords:** laser-induced phase transitions, GeTe, ablation

## Abstract

Realizing the full potential of optical actuation for high-speed phase-change radio-frequency (RF) switches requires a shift to single-pulse operation. This work presents a systematic investigation of reversible phase transitions in GeTe thin films induced by single 10 ns laser pulses, utilizing spatially resolved characterization techniques, including atomic force microscopy (AFM) and micro-spectroscopy. Precise laser fluence windows for crystallization (12.7–16 mJ/cm^2^) and amorphization (25.44–41.28 mJ/cm^2^) are established. A critical finding is that the amorphization process is governed by rapid thermal accumulation, which creates a direct trade-off between achieving the phase transition and avoiding detrimental surface morphology. Specifically, we observe that excessive energy leads to the formation of laser-induced ridges and ablation craters, which are identified as primary causes of device performance degradation. This study elucidates the underlying mechanism of single-pulse-induced phase transitions and provides a practical processing window and design guidelines for developing high-performance, optically actuated GeTe-based RF switches.

## 1. Introduction

As a chalcogenide phase-change material, GeTe exhibits pronounced contrasts in electrical resistivity and optical reflectance between its crystalline and amorphous states [1,2,3]. While traditional phase-change materials like Ge_2_Sb_2_Te_5_ (GST) have been predominant in the non-volatile memory landscape [3], GeTe has emerged as a superior candidate for reconfigurable radio-frequency (RF) devices [4,5,6]. Its advantages include a higher crystallization temperature, which ensures enhanced thermal stability, an exceptionally high electrical resistivity contrast (>10^6^) crucial for a high ON/OFF ratio, and superior hole mobility that benefits the ON-state insertion loss [7]. These characteristics are particularly desirable for RF switches, where signal integrity and power handling are paramount.

The actuation method for these phase-change devices is equally crucial. Laser processing has gained significant industrial relevance as a powerful material modification tool, offering unparalleled advantages in non-contact operation, spatial precision, energy efficiency, and outstanding process repeatability [8,9]. For RF systems, optical actuation presents a compelling alternative to electrical signaling by eliminating the need for integrated electrodes, thereby mitigating parasitic capacitance and enabling more flexible device architectures [10].

The fundamental kinetics of GeTe support rapid phase transitions. Extensive research has confirmed that crystallization and amorphization can be achieved on the nanosecond scale using laser pulses [11,12,13,14,15]. For instance, studies have shown that the phase transition can be completed within tens of nanoseconds [11,15]. However, when translating this fundamental speed into functional RF switches, the state of the art in optical actuation has yet to fully capitalize on it. Demonstrations, such as the work by Charlet et al. utilizing laser pulses with durations of hundreds of nanoseconds to microseconds, result in switching speeds comparable to electrical switches [16], thus failing to exploit the inherent speed advantage of photonic control [10,17,18].

This performance gap is further widened by the research focus within the GeTe community. Driven largely by the demands of multi-level memory applications, significant research efforts have been directed towards understanding and exploiting multi-pulse and gradual phase transition mechanisms [19,20,21,22,23,24], with single-pulse switching receiving considerably less attention. This prevailing trend, however, is misaligned with the operational paradigm of a high-speed RF switch. Unlike memory that may benefit from multi-level programming, an RF switch is a binary device requiring a rapid and definitive phase transition. A single-pulse paradigm is, therefore, not merely an option but a necessity for achieving ultimate switching speed, operational simplicity, and robustness. Our work addresses this critical gap by focusing on the phase transition induced by a single ~10-nanosecond laser pulse. We posit that by drastically reducing the pulse duration from the microsecond to the nanosecond regime, we can unlock the potential for switching speeds orders of magnitude faster than the current optically actuated state of the art. The investigation into the fundamental single-pulse phase control at this timescale, and its correlation with the resulting phase states and morphology, constitutes the key novelty of our study.

In this study, we systematically investigate the effects of varying fluence from a single 532 nm nanosecond laser pulse on the crystallization and amorphization processes of GeTe thin films. Using a focused Gaussian beam and characterization techniques including reflectance monitoring and AFM, we explore the mechanisms underlying these ultrafast laser-induced phase transitions. Our goal is to identify the precise laser fluence windows for controlled phase transitions and to understand the concomitant morphological changes. Based on these findings, we discuss potential strategies to mitigate ablation and improve the quality of phase transitions, paving the way for the development of future high-speed, optically actuated GeTe-based RF switches.

## 2. Materials and Methods

### 2.1. Experimental Setup

**Figure 1:**
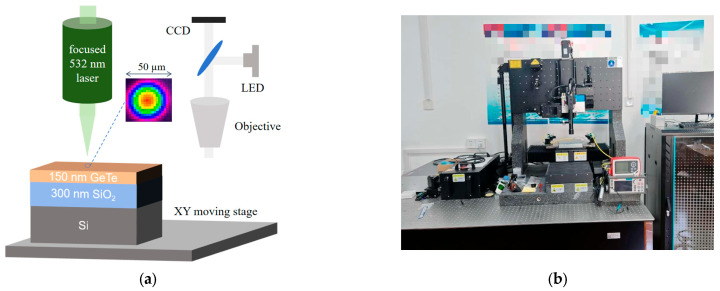
Scheme of the laser experimental setup: (**a**) schematic diagram; (**b**) machining platform.

A 150 nm thick GeTe thin film (Zhongnuo New Material, Beijing, China) was deposited by magnetron sputtering (60 W, 0.4 Pa) on a 300 nm SiO_2_ underlayer, which serves as an effective thermal insulation layer. The selection and thickness of this insulation layer are of paramount importance for controlling the thermal dynamics during the phase transition process. Prior to laser irradiation, the samples underwent a standardized cleaning procedure to remove surface contaminants: sequential ultrasonication in acetone, ethanol, and deionized water (5 min each), followed by drying under a gentle nitrogen stream. A schematic diagram of the experimental setup and a corresponding photograph are shown in Figure 1a,b, respectively. The irradiation source was a 532 nm pulsed laser (Guangzhi Technology Co., Ltd, Wuhan, China), focused to a Gaussian spot with a D4σ beam diameter of approximately 50 μm. The samples were mounted on a computer-controlled XY translation stage, and their position was monitored in real-time using a charge-coupled device (CCD) camera. Dedicated software (Femto Easy STAR) synchronized the laser output with the stage movement. The laser pulse energy and beam profile were characterized using a laser power meter (Thorlabs) and a beam profiler, respectively.

**Figure 2:**
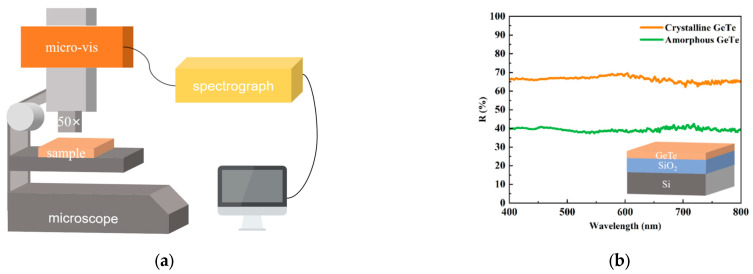
Schematic diagram and results of reflectance measurement: (**a**) schematic diagram; (**b**) reflectivity of as-deposited GeTe and annealed crystalline GeTe.

The pronounced contrast in optical reflectivity serves as a robust and well-established indicator of the crystalline-to-amorphous phase transition in GeTe and related phase-change materials [12,25]. We constructed a microscale reflectance measurement system by integrating spectroscopic modules into an optical microscopy, which can be used to quantify the reflectance of a wide spectrum ranging from 400 to 800 nm within micrometer-scale regions, as shown in Figure 2a. The measured reflectance of crystalline and amorphous GeTe is shown in Figure 2b. GeTe exhibits an obvious reflectance contrast between the two states: approximately 70% for the crystalline phase versus about 40% for the amorphous phase. Phase identification was primarily based on the characteristic reflectivity contrast between amorphous and crystalline GeTe, as measured by micro-spectroscopy.

### 2.2. Theoretical Analysis

Laser-induced phase transition in GeTe primarily originates from the photothermal effect. Unlike the non-thermal phase transition mechanisms potentially induced by femtosecond pulses, the phase transition driven by nanosecond pulses is primarily governed by thermal effects [26,27]. This process follows a classical thermodynamic pathway, wherein the laser heats the material above its crystallization or melting temperature, followed by a phase transition achieved through thermal conduction. Although the spatial control precision of nanosecond lasers is inferior to that of femtosecond lasers, they offer advantages in terms of lower cost and easier integration. Upon reaching the phase transition temperature threshold, GeTe undergoes a series of structural changes. When crystalline GeTe is heated above its melting point (~998 K), it transforms into a liquid state. After the pulse terminates, this molten state is rapidly quenched by the substrate at an estimated rate of ~10^9^–10^11^ K/s, solidifying into an amorphous phase [28]. Conversely, when amorphous GeTe is heated above its crystallization temperature (473–673 K), its atoms gain sufficient kinetic energy to diffuse and rearrange, facilitating nucleation and subsequent crystal growth.

To model the laser-induced crystallization and amorphization processes in GeTe, we performed simulations using the COMSOL Multiphysics software (Version 6.1, Stockholm, Sweden). In this model, the laser energy incident on the material surface causes a rapid temperature increase. The resulting energy transfer is governed by thermal radiation to the surroundings, heat conduction into the material bulk, and natural convection in the adjacent air. Accordingly, an energy conversion model accounting for these phenomena can be established [29].

(1) $$\rho C_{p}\frac{\partial T}{\partial t}-k\times ∆T+h(T_{0}-T)+\varepsilon \sigma (T_{0}^{4}-T^{4})=Q$$

Here, $\rho ,\;C_{p}$, k, h, ε and σ represent the material’s density, specific heat capacity, thermal conductivity, convective heat transfer coefficient, emissivity, and the Stefan–Boltzmann constant, respectively.

The incident laser beam undergoes rapid attenuation upon penetration into GeTe, where absorption follows the Beer–Lambert law [19]. The laser power density within the phase-change film decays exponentially; I(z) represents the depth-dependent laser irradiance:

(2) $$I(z)=\left(1-R\right)\times I_{0}\times exp(-\alpha z)$$

where $I_{0}$ is incident irradiance at the surface (z = 0), α is absorption coefficient of GeTe (cm^−1^), and z is depth coordinate (μm).

In the radiation beam module of the absorption medium, the light spot type is set as a Gaussian spot; the incident beam power density deposited on the thin-film surface is given by

(3) P=(E×S)/τ×g(t)×(1−R)

where E is the laser fluence, S is the irradiated area, R is the surface reflectivity, and g(t) represents the laser temporal profile such as rectangular or Gaussian pulses.

In this specific experiment, the experimental power measured by a power meter was used to derive the single-pulse energy, ultimately yielding the deposited beam power.

**Figure 3:**
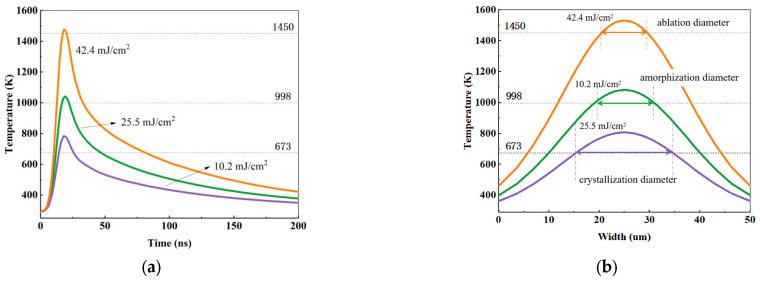
Simulated temperature evolution: (**a**) time-resolved temperature profiles at the center (10 μm-diameter region) of the GeTe surface under three laser fluence levels; (**b**) cross-sectional temperature distributions across the GeTe surface at t = 20 ns for the corresponding fluence conditions.

COMSOL thermal simulations of crystalline and amorphous states of GeTe under varying laser fluence levels reveal dynamic surface temperature evolution (Figure 3); the material parameters are shown in Table 1. Figure 3a demonstrates transient thermal profiles within a 10 µm surface region, where rapid heating (≤20 ns) followed by gradual cooling completes the thermal cycle within 200 ns. Horizontally dashed lines mark critical thresholds for ablation (1450 K), amorphization (998 K), and crystallization (673 K). Corresponding cross-sectional temperature distributions in Figure 3b enable quantification of the diameters of the phase-changed regions via critical isotherm propagation (e.g., 1450 K). These simulated diameters are quantitatively validated against experimental measurements in Section 3.

**Figure 4:**
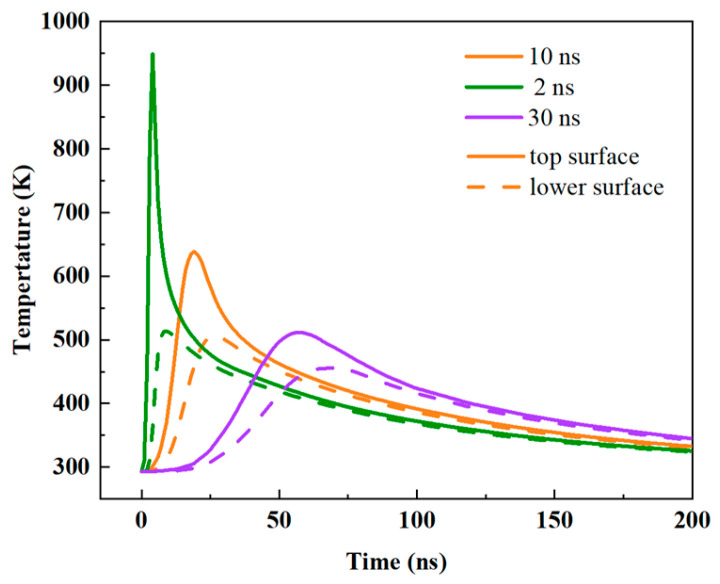
Temperature variations of crystalline GeTe under different pulse widths at 30 mW laser power. The solid lines represent the top surface and the dashed lines (green and purple) represent the lower surface.

In this experiment, a laser pulse width of 10 ns was employed. The nucleation and crystalline growth processes of GeTe occur on timescales ranging from nanoseconds to tens of nanoseconds. A 10 ns laser pulse provides sufficient duration for nucleus formation and short-range atomic diffusion, facilitating the formation of uniform, high-quality crystals. Concurrently, its brevity is adequate to prevent excessive grain growth and thermal diffusion. As shown in Figure 4, at the same laser fluence, a shorter pulse width can achieve a higher peak temperature. However, this induces a greater temperature gradient across the GeTe film, leading to inferior phase transition homogeneity, a narrower process window, and more challenging control. Conversely, a 30 ns laser pulse may result in secondary crystallization due to an insufficient cooling rate. The 10 ns timescale is well-matched with the intrinsic crystallization kinetics (nucleation and growth) of GeTe, thereby contributing to high-performance reversible phase transitions.

## 3. Results and Discussion

### 3.1. Amorphization Behavior

**Figure 5:**
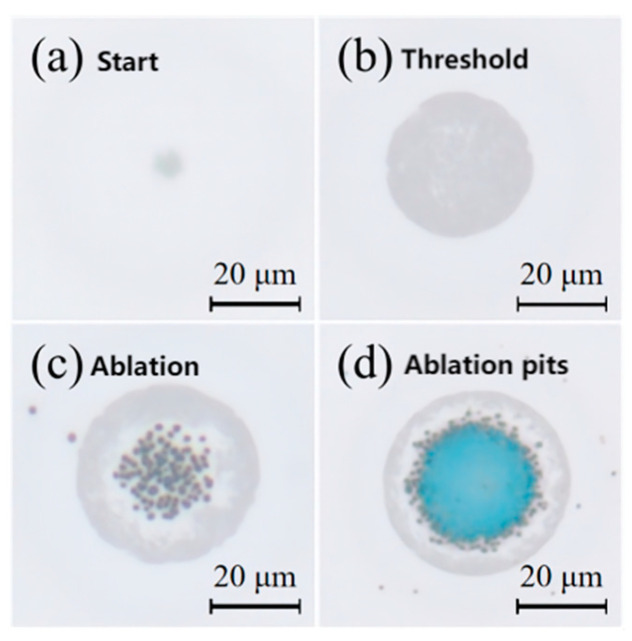
Amorphization phenomena of GeTe observed under the microscope: (**a**) initiation of amorphization; (**b**) expansion of the amorphized region; (**c**) formation of ablation particles; (**d**) creation of ablation pits and appearance of the blue SiO_2_.

**Figure 6:**
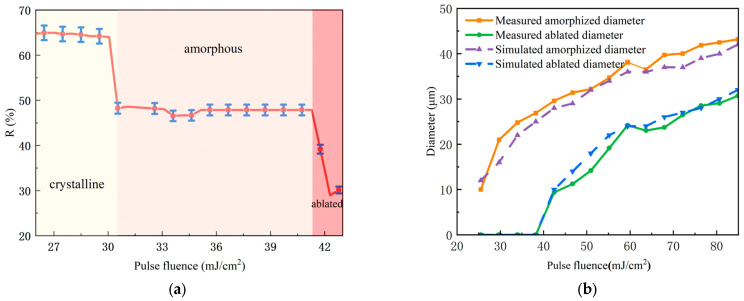
Variations in the amorphized spot with increasing laser fluence: (**a**) reflectivity changes, where the red dashed line represents the reflectivity of annealed crystalline GeTe and the blue dashed line corresponds to the reflectivity of as-deposited amorphous GeTe; (**b**) changes in the amorphization diameter and ablation diameter, as obtained from both simulation and experimental measurements.

Laser irradiation induced a series of amorphized spots on the initially crystalline GeTe films. Progressive increases in laser fluence revealed four distinct stages of surface evolution, as illustrated in Figure 5. The corresponding changes in reflectance during amorphization were characterized by micro-spectroscopy (Figure 6a), which delineated the following stages:

1. Initial stage (<25.44 mJ/cm^2^): No significant morphological changes were observed.
2. Amorphization stage (25.44–41.28 mJ/cm^2^): This higher fluence range delivered enough energy to completely melt the GeTe film (temperature > 998 K). Gray circular spots emerged, indicating phase transition (Figure 5a). The diameter of amorphized regions increased with laser power, attributed to the radial threshold gradient of the Gaussian energy profile (Figure 5b). The reflectivity of the amorphized region decreased to 47% ± 2%. The simulated laser fluence threshold was verified by micro-spectroscopic measurements. The detected reflectance change occurred at a slightly lower fluence than that needed for an optically visible spot, as the spectroscopic method is more sensitive to incipient phase transitions. In the fluence of 38 mJ/cm^2^, the maximum ablation-free amorphized diameter reached 25 μm.
3. Ablation stage (>41.28 mJ/cm^2^): The spot coverage approached 50 μm, with the expansion rate decreasing as ablation initiated (Figure 5c). Further increasing the fluence causes the central temperature to exceed the ablation threshold, leading to observable ablation. The resulting surface became populated with particulates, consequently enhancing diffuse scattering and reducing the measured reflectance to around 30%. Ultimately, at the highest fluences, the blue SiO_2_ substrate was exposed, confirming the complete ejection of GeTe from the central region.

Based on the theoretical analysis presented in Section 2, the evolution and distribution of the surface phase transition temperature were derived. Amorphization initiates when the temperature exceeds 998 K [19], while material ablation occurs at temperatures exceeding 1450 K. Figure 6b compares these simulation results with experimental measurements, demonstrating excellent agreement between the experimentally measured curve and the finite element method (FEM) simulation results. The diameter of the amorphization region exhibits a strong dependence on laser fluence, with a marked nonlinear relationship.

**Figure 7:**
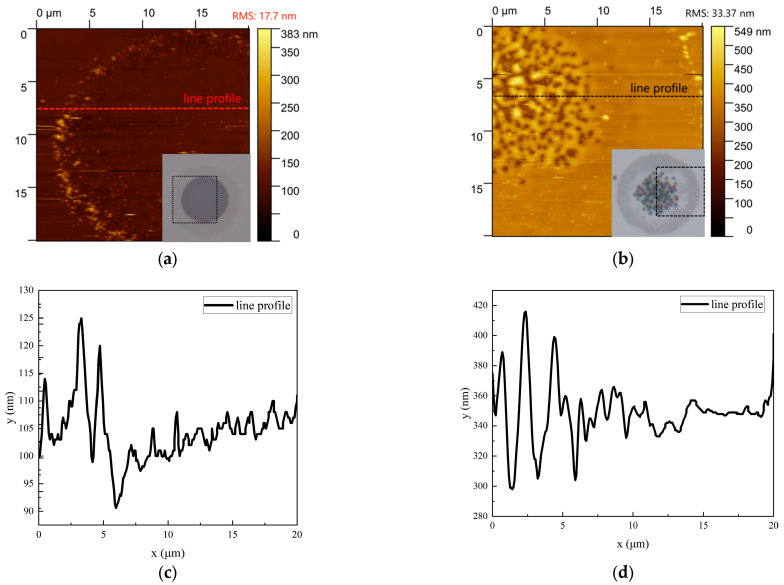
AFM images of the amorphous (38 mJ/cm^2^) and ablated (45 mJ/cm^2^) regions formed by single-pulse irradiatio: (**a**) amorphous state, RMS:17.7 nm; (**b**) ablated state, RMS:33.37 nm; (**c**) Roughness line profiles along the dashed lines in (**a**). (**d**) Roughness line profiles along the dashed lines in (**b**).

AFM 2D topography characterization was conducted on amorphized and ablated regions, as illustrated in Figure 7. The AFM images were quantitatively analyzed using Gwyddion 2.58 software. The line profiles in Figure 7c,d, which were extracted along the corresponding lines in Figure 7a,b, respectively, reveal the cross-sectional height distribution of the surface structures. In Figure 7a, the amorphized area exhibits distributed small spikes surrounded by peripheral ridge structures. The Gaussian beam profile results in lower fluence at the periphery. Driven by thermal gradients and the Marangoni effect [24], the resulting surface-tension imbalance propels molten material toward cooler regions, thereby forming ridge structures. Height variations in the peripheral ridges are attributed to film preparation defects. These laser-induced protrusions, formed during high-energy pulse amorphization, degrade electrode contact performance and could emerge as critical failure factors in future device fabrication.

When laser fluence exceeds the ablation threshold of GeTe films, ablation occurs—characterized by the formation of ablation particles and pits (Figure 5c). At this time, the laser fluence is 45 mJ/cm^2^, and the diameter of the central ablation area is about 15 μm, which is consistent with the ablation diameter in the simulation. The central region undergoes initial disruption, generating ablation particles as shown in Figure 7b: a substantial increase in RMS roughness was observed. Central protrusions rise approximately 60 nm above the surface with subsurface depressions approximately 40 nm deep. Molten GeTe aggregates into protrusions while partially ejecting as droplets—thereby contributing to particulate formation. As laser fluence increases, peripheral regions subsequently reach melting temperatures. With further temperature increases, vaporization and material removal ensue, ultimately forming ablation pits (Figure 5d). Here, vapor recoil pressure drives molten material to expand radially outward, yielding raised rims around the pits. This ablation not only significantly increases RMS roughness but also alters GeTe composition. The irreversible material removal represents permanent film damage, ultimately leading to material failure.

### 3.2. Crystallization Behavior

**Figure 8:**
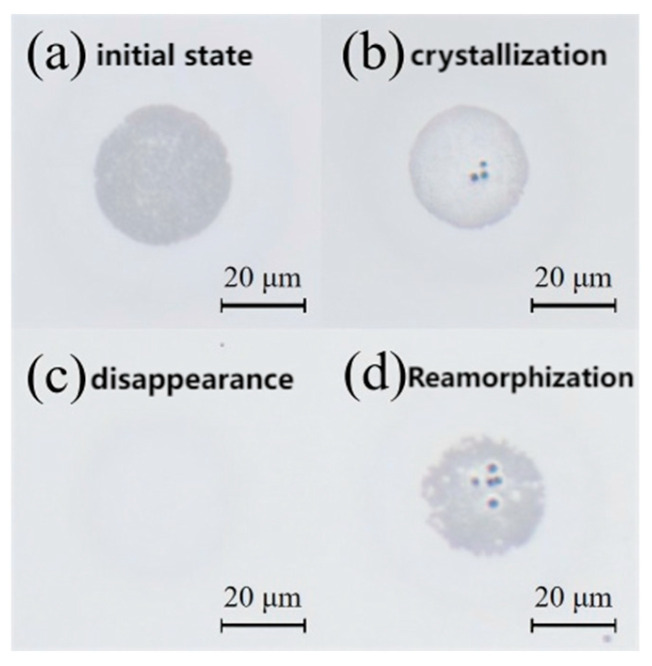
Single-pulse crystallization of pre-amorphized GeTe (amorphized at 38 mJ/cm^2^): (**a**) no fluence; (**b**) 8.5 mJ/cm^2^; (**c**) 12.7 mJ/cm^2^; (**d**) 16 mJ/cm^2^.

**Figure 9:**
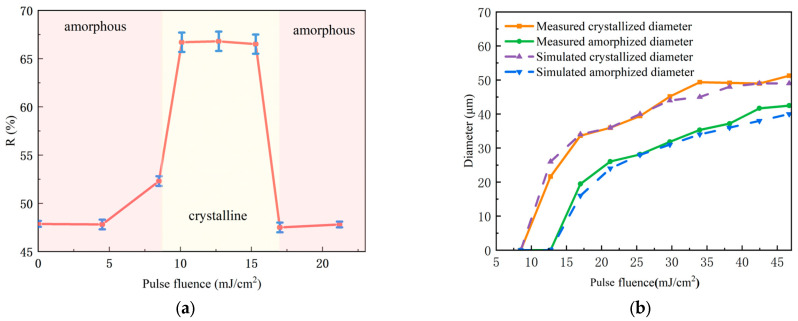
Variations in the crystallized region: (**a**) reflectivity changes under different pulse fluences; (**b**) changes in the diameters of the crystallized and amorphized regions with increasing pulse fluence, as obtained from both simulation and experimental measurements.

To investigate the crystallization behavior of GeTe thin films, a pulsed laser with a fluence of 38 mJ/cm^2^ was initially employed to irradiate the GeTe thin film, creating an amorphized region with a diameter of 25 μm. The crystallization behavior of these amorphized regions was then studied. As depicted in Figure 8, four distinct evolution stages emerge; Figure 9a demonstrates reflectance transitions: as fluence increases, surface reflectance first reaches crystalline levels then reverts to amorphous-state levels.

1. Initial state: When there is no pulse excitation or the pulse energy is lower than 8.5 mJ/cm^2^, the original amorphous state is maintained.
2. Crystallization (8.5–15 mJ/cm^2^): Within this range, the laser energy deposition is sufficient to raise the film temperature above the crystallization temperature (~673 K) but below the melting point (~998 K). When laser fluence exceeds 8.5 mJ/cm^2^, the degree of amorphization weakens and the reflectivity increases; when laser fluence exceeds 10.2 mJ/cm^2^, the amorphous spot completely disappears. At this point, the surface reflectivity of GeTe film is about 67%, which has been crystallized. This energy input provides the necessary thermal activation for atomic rearrangement, allowing for the growth of crystallized nuclei from the amorphous matrix through a solid-phase crystallization mechanism.
3. Reamorphization state (>15 mJ/cm^2^): Further increasing fluence causes the central region temperature to exceed the amorphous threshold, leading to reamorphization, and the reflectivity level of GeTe film returns to amorphous state.

**Figure 10:**
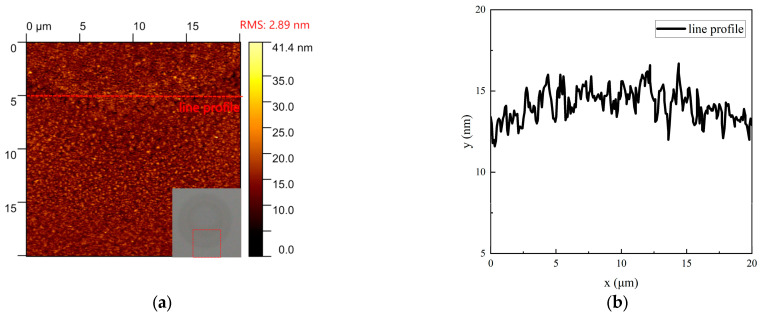
Recrystallization: (**a**) AFM images of the amorphous (38 mJ/cm^2^) and crystallized (10.2 mJ/cm^2^) regions formed by sequential single-pulse irradiation, RMS: 2.89 nm; (**b**) Roughness line profiles along the dashed lines in (**a**).

The spot diameter evolution versus laser fluence in Figure 9b shows crystalline/amorphous diameters from simulations and experiments, exhibiting trends consistent with Figure 6b. AFM analysis (Figure 10) indicates a ~5 nm surface height after recrystallization, showing collapsed morphology with reduced protrusions. Volume shrinkage during crystallization promotes inward collapse and surface planarization [16].

**Figure 11:**
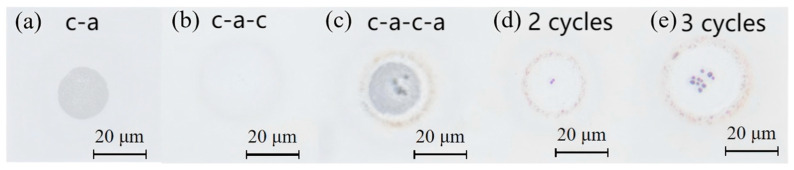
Phase transition states under the microscope: (**a**) first amorphized state; (**b**) first transition cycle (amorphization → crystallization); (**c**) second amorphized state after two amorphization and one crystallization cycles; (**d**) second transition cycle (two amorphization + two crystallization); (**e**) third transition cycle showing particulate formation (three amorphization + three crystallization).

Cyclic phase transition was further investigated. While single-phase transition caused no damage (Figure 11), repetitive induction at identical power induced cumulative edge effects. Irreversible damage occurred during the recrystallization process. As shown in Figure 11e, ablation particles were formed after three cycles, which posed a great challenge to the cycle durability of GeTe. In order to improve the durability of GeTe, this can be mitigated by optimizing the laser beam profile (e.g., using a flat-top beam to alleviate edge effects) and by improving the film quality to reduce defect formation, thereby suppressing the development of surface spikes during laser irradiation.

In this study, we developed a multiphysics-coupled model integrating laser energy and phase transition temperature to calculate the laser energy required for GeTe thin-film phase transition under Gaussian pulse excitation, with experimental verification. Compared to the existing literature (Table 2) that predominantly employs multi-pulse excitation methods, our approach using a 532 nm laser with narrower pulse width in reference [12] achieves more efficient laser energy utilization. While the Gaussian spot configuration in this study reduces phase transition energy requirements, morphological characterization and cyclic testing demonstrate that the lower energy consumption compromises GeTe’s phase transition stability and increases failure risks.

## 4. Conclusions

This study demonstrates selective and reversible phase transitions in 150 nm GeTe films induced by single 10 ns Gaussian laser pulses, where non-volatile amorphization and crystallization are controlled precisely via laser fluence. The principal conclusions are summarized as follows:

1. The threshold fluences were quantitatively determined: recrystallization occurs at 8.5 mJ/cm^2^, while melt-quenching amorphization requires 25.4 mJ/cm^2^, consistent with coupled thermal–optical simulations and time-resolved reflectometry.
2. Morphological degradation—including increased roughness, nanoscale protrusions, and cyclical ablative loss—currently limits endurance. This underscores the material-level challenge in achieving high-cycle operability.
3. For the realization of reliable optically addressable phase-change devices, future efforts should concentrate on (i) optimizing GeTe stoichiometry and interfacial adhesion, (ii) developing engineered beam profiles with flat-top intensity distributions, and (iii) investigating the early stages of degradation to implement predictive failure mitigation strategies.

## Figures and Tables

**Table 1:** Material parameters.

	SiO_2_	GeTe_a	GeTe_c
Cp [J/(kg·K)]	730	278	252
Rho [kg/m^3^]	2203	5600	5910
Thermal conductivity [W/(m·K)]	1.38	3.08	3.08
Absorption coefficient [1/m]	1 × 10^−3^	5.71 × 10^7^	6.64 × 10^7^
Conductivity [S/m]	0	0.64	3 × 10^5^
Relative dielectric constant	4.2	18	18
Transition temperature [K]	-	473–673	998
Erosion temperature	-	-	1450

**Table 2:** Comparison of energy thresholds for phase transition in GeTe films under different laser parameters.

Document	GeTe Thickness	Substrate	Laser Parameters	Crystallization	Amorphization
[23]	75 nm	Si	248 nm, 20 ns, pump-probe setup	26 mJ/cm^2^, 5 pulses	112 mJ/cm^2^
[25]	60 nm	Si	248 nm, 20 ns, pump-probe setup	11–14 mJ/cm^2^ >5 pulses	162–182 mJ/cm^2^
[12]	200 nm	Al_2_O_3_	248 nm, 30 ns, a KrF excimer laser	90 mJ/cm^2^	180 mJ/cm^2^
[17]	100 nm	SiO_2_	532 nm, 13 ns, Top hat	47.6 mJ/cm^2^	70 mJ/cm^2^
This work	150 nm	SiO_2_	532 nm, 10 ns, Gaussian	8.5–15 mJ/cm^2^	25.4 mJ/cm^2^

## Data Availability

The original contributions presented in this study are included in the article. Further inquiries can be directed to the corresponding author.

## Abbreviations

The following abbreviations are used in this manuscript:

GeTe	Germanium telluride
AFM	Atomic Force Microscope
CCD	Charge-Coupled Device
RF	Radio-Frequency
GST	GeSbTe
UV	Ultraviolet
FEM	Finite Element Method
RMS	Root-Mean-Square Error
